# Clinical Indications to Germectomy in Pediatric Dentistry: A Systematic Review

**DOI:** 10.3390/ijerph19020740

**Published:** 2022-01-10

**Authors:** Marta Mazur, Artnora Ndokaj, Beatrice Marasca, Gian Luca Sfasciotti, Roberto Marasca, Maurizio Bossù, Livia Ottolenghi, Antonella Polimeni

**Affiliations:** 1 Department of Oral and Maxillofacial Sciences, Sapienza University of Rome, Via Caserta 6, 00161 Rome, Italy; marta.mazur@uniroma1.it (M.M.); beatrice.marasca@uniroma1.it (B.M.); gianluca.sfasciotti@uniroma1.it (G.L.S.); maurizio.bossu@uniroma1.it (M.B.); livia.ottolenghi@uniroma1.it (L.O.); antonella.polimeni@uniroma1.it (A.P.); 2 Pediatric Dentistry Unit, Head and Neck Integrated Department, AOU Policlinico Umberto I of Rome, Viale Regina Elena 287/b, 00161 Rome, Italy; r.marasca@policlinicoumberto1.it

**Keywords:** germectomy, oral surgery, clinical indications, oral health, dentistry, systematic review

## Abstract

Germectomy is a procedure often required in patients at developmental age. It is defined as the surgical removal of the third molar at a very specific stage of development. The aim of this study was to systematically analyze the literature in terms of clinical indications for germectomy in patients at developmental age. Literature searches were performed using PubMed, Google Scholar, Cochrane Library and Scopus from 1952 to 30 June 2021. The study protocol was registered after the screening stage (PROSPERO CRD42021262949). The search strategy identified 3829 articles: 167 from PubMed, 2860 from Google Scholar, 799 from Cochrane Library and 3 from Scopus. Finally, eight full-text papers were included into the qualitative analysis. Based on the included studies, clinical indications for germectomy were mainly related to orthodontic causes, infectious and cariogenic causes and prophylaxis. Based on these results, it is not possible to present evidence-based clinical indications for germectomy in patients at developmental age. Clinical trials on this subject focused specifically on patients at developmental age are awaited.

## 1. Introduction

Demirjian’s classification system distinguishes the third molar development based on shape and it divides the entire process into eight stages: A–D, which represent the crown formation from the appearance of the cusps to the crown completion, and stages E–H, which represent root formations from radicular bifurcation to apical closing [1]. In this scenario, it is possible to provide a definition of germectomy of the third mandibular molar: it is the surgical removal of the third molar at the developmental stage of B, C and D.

Moreover, based on the retrospective radiographic study by Jung and Cho, who analyzed the panoramic radiographs of 2490 patients aged between 6 and 24, it is possible to strongly correlate the developmental stages of third molars to the chronological age of patients. In fact, the stages B, C and D of mandibular third molars are correlated to an age range from 10 to 16 in most of the cases [2].

A very recent update of a Cochrane review firstly published in 2012, aiming to find out whether asymptomatic and disease-free wisdom teeth should be removed or left alone and checked at regular intervals, in a large age group of patients, ranging from teenagers to adults, showed that no evidence is present about clinical indications to prophylactic removal. Moreover, based on this review, it is worth saying that pediatric oral surgery is not presented in the literature in an autonomous manner, and in fact, developmental patient problems are often treated alongside those of adult patients, leading to data loss and confusion in epidemiological representation and indications to treatment [3].

Treatment of a developmental patient can often be multidisciplinary and may involve several specialists, such as a pediatric dentist, orthodontist and oral surgeon. Providing clear indications for a germectomy can be of great help to all these specialists in order to better manage the therapeutic plan for the young patient. Ideally, it would be advisable that, e.g., after the end of orthodontic treatment in a teenager, they could have a stable dental situation without the need for further intervention.

The aim of this systematic review was to comprehensively analyze the literature in order to provide clinical indications to germectomy in a population at developmental age.

## 2. Materials and Methods

This systematic review was conducted in accordance with the Preferred Reporting Items for Systematic Reviews and Meta-Analyses (PRISMA) statement and the guidelines from the Cochrane Handbook for Systematic Reviews of Interventions [4]. The study protocol was registered after the screening stage (PROSPERO CRD42021262949).

### 2.1. Eligibility Criteria

The following inclusion criteria were applied for this systematic review: (a) randomized controlled trials (RCTs); (b) clinical trials; (c) case-control studies; (d) cohort studies; (e) cross-sectional studies; (f) clinical human studies based on human dentate. Studies published in English, French, German, Spanish, Polish and Albanian were included. Broad inclusion criteria have been used to be as sensitive as possible. The followings were the exclusion criteria: (a) in-vitro RCTs; (b) lack of effective statistical analysis; (c) abstract and author debates or editorials.

### 2.2. Search Strategy and Study Selection

Literature searches of free text and MeSH terms were performed using PubMed, Google Scholar, Cochrane Library and Scopus from 1952 to 30 June 2021. All searches were conducted using a combination of subject headings and free-text terms. The final search strategy was determined through several pre-searches. The keywords used in the search strategy were as follows: (indications[All Fields] AND ((“molar, third”[MeSH Terms] OR (“molar”[All Fields] AND “third”[All Fields]) OR “third molar”[All Fields] OR (“third”[All Fields] AND “molar”[All Fields])) OR (“molar, third”[MeSH Terms] OR (“molar”[All Fields] AND “third”[All Fields]) OR “third molar”[All Fields] OR (“wisdom”[All Fields] AND “tooth”[All Fields]) OR “wisdom tooth”[All Fields]) OR ((“molar, third”[MeSH Terms] OR (“molar”[All Fields] AND “third”[All Fields]) OR “third molar”[All Fields] OR (“third”[All Fields] AND “molar”[All Fields])) AND germ[All Fields])) AND extraction[All Fields] AND (“child”[MeSH Terms] OR “child”[All Fields] OR “children”[All Fields]) AND (“paediatric dentistry”[All Fields] OR “pediatric dentistry”[MeSH Terms] OR (“pediatric”[All Fields] AND “dentistry”[All Fields]) OR “pediatric dentistry”[All Fields])).

Reference lists of primary research reports were cross-checked in an attempt to identify additional studies. Following the inclusion criteria, two authors (M.M. and A.N.) independently selected the literature by reading the titles and abstracts. The full text of each identified article was then read to determine whether it was suitable for inclusion. Disagreements were resolved through consensus or by discussion with a third author (R.M.).

### 2.3. Data Collection

For each eligible study, data were independently extracted by two authors (M.M. and A.N.) and examined by the third author (R.M.) by creating a piloted spreadsheet and comparing them through it, in accordance with the Cochrane Collaboration guidelines. In cases of missing data, M.M. contacted the corresponding author of the related research via email and excluded those for which no reply was received.

### 2.4. Data Items

The following data items were recorded: author, year, study type (randomized controlled trial, case-control cohort, cross-sectional), population (number of subjects and mean age and/or standard deviation), number of patients at developmental age, percentage of female patients, study setting (hospital, private practice, university clinic), intervention (extraction), comparison/no-intervention, clinical indication to extraction and any outcome, if present.

### 2.5. Quality Assessment

According to the PRISMA statements, the evaluation of the methodological quality gives an indication of the strength of evidence provided by the study because methodological flaws can result in biases. For the randomized clinical trials, according to the Jadad scale [5], this procedure provides a total score that can range from 0 to 5, where 0 is a low-quality study and 5 is the highest possible quality. A trial is considered to have a good quality when it gets a score of at least 3. For case-control and cohort studies, according to the Newcastle–Ottawa scale (NOS) [6], the possible quality assessment score ranges from zero to nine points, with a high score indicating a good quality study.

### 2.6. Risk of Bias in Individual Studies

Selection bias (retained allocation concealment), performance and detection bias (blinding of participants and operators), attrition bias (patient dropout, wash-out period of cross over trials and missing values or participants and too short duration of follow-up) and reporting bias (selective reporting, unclear eliminations and missing results) were recorded, evaluated and allocated according to Cochrane guidelines [4].

## 3. Results

### 3.1. Study Selection

**Figure 1:**
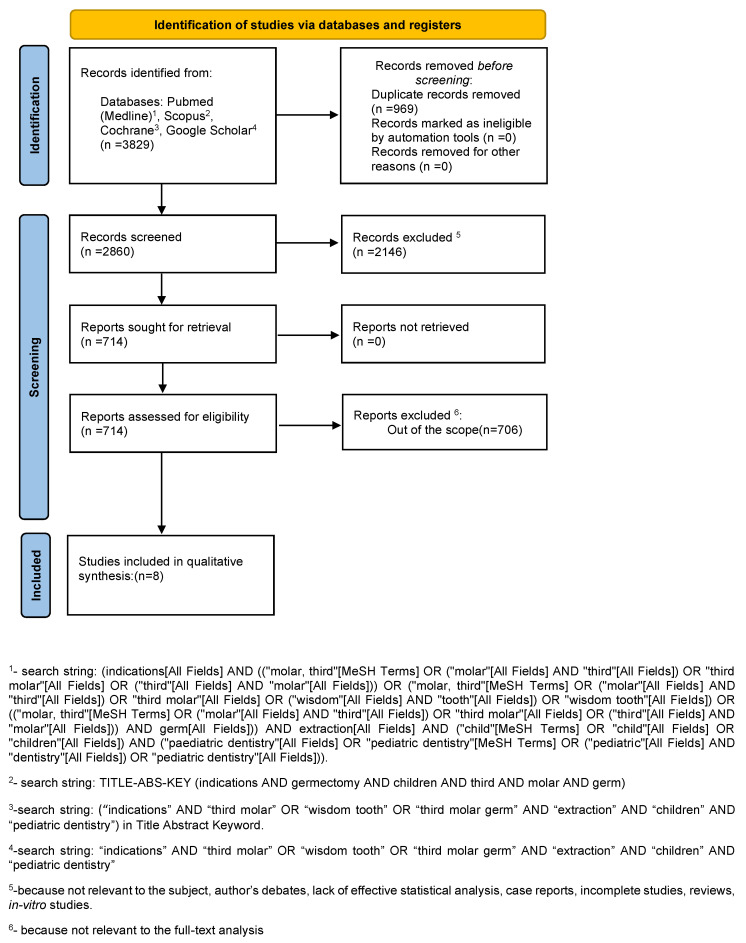
Flowchart of the search.

The search strategy identified 3829 potential articles: 167 from PubMed, 3 from Scopus, 799 from Cochrane Library and 2860 from Google Scholar. After removal of duplicates, 2860 articles were analyzed. Subsequently, 2146 papers were excluded because they did not meet the inclusion criteria. Of the remaining 714 papers, 706 were excluded because they were not relevant to the subject of the study. The remaining eight papers were included in the qualitative synthesis (Figure 1). Table 1 summarizes the characteristics of each of the eight included studies.

### 3.2. Study Characteristics

The included studies (Table 1) were published between 1995 and 2017 and consisted of one randomized controlled trial, one case control study and seven cohort studies (four retrospective and three prospective). Total sample size was 4640 participants (range: 20–1763), and a subset of 879 subjects was at developmental age range. Overall, 48.2% were female patients.

### 3.3. Quality Assessment

According to the Jadad scale for RCT (n = 1) [7], the authors evaluated the quality of one clinical trial included in the qualitative synthesis, based on five questions that analyze the randomization process, the experimental blinding, and the dropout rate, i.e., the patients lost to follow-up. In the evaluation of the quality of RCTs the total score of this study was 3, indicating a good quality study (Table 2).

According to the Newcastle–Ottawa scale (NOS) for case-control studies (n = 1) [8] and cohort studies (n = 6) [9,10,11,12,13,14], the authors evaluated the qualities of all included studies based on object selection, comparability and exposure. A star was described as an appropriate entry, with each star representing one point. The possible quality assessment score ranged from zero to nine points, with a high score indicating a good quality study. In the evaluation of the quality of the case-control study, the total score was 7, indicating a high-quality study (Table 3). With regards to the quality of the cohort studies, the total score of four of the included studies was greater than or equal to 6, indicating high-quality studies, while for the remaining three studies, the total score was 5, indicating good quality (Table 4).

**Table 1:** Study characteristics.

Author	Year	Study Type	Population (Age and n)	Mean Age/Age Range and/or σ	Developmental Age Patients (Age and n)	Female %	Setting (University Clinic, Dental Clinic)	Intervention (Extraction) and n	Comparison-No Intervention (If Present, State: No Extraction or Other) and n		Clinical Indication to Extraction		Any Outcome, If Present
Adeyemo [9]	2008	cohort	15–92 n = 1763	33.74 ± 13.3	10–19 n = 62	58%	University of Lagos, Nigeria	506 surgical ex	1257 no ex	506 ex	caries n.34; pericoronitis n.28		-
Chiapasco [10]	1995	cohort	9–67 n = 868	Group A: 9–16 y Group B: 17–23 y Group C: >24 y	9–16 n = 254	53%	University of Milan, Italy	254 surgical ex			orthodontic indication		Complications: Group A 9–16y 2.6%; B 17–24 y 2.8%; C >24 y 7.4%
D’Angeli [11]	2021	cohort	11–17 n = 25	15.44 ± 2.06 y	11–17 n = 25	40%	Sapienza University of Rome, Italy	46 surgical ex			orthodontic indication		Complications: 4.2%, no association among gender, Winter’s class, germ development
Monaco [12]	2016	cohort	12–20 n = 134	15		49%	University of Bologna, Italy	218 surgical ex			orthodontic indication		Delayed infection: 20 (9.2%), 16/20 Ganss Ratio < 0.5
Werkmeister [8]	2005	case-control	11–81 n = 616	316 test, mean a:33.3; 300 control, mean a: 26.7		test group: 25.3 %, control group 47%	University Hospital of Muster, Switzerland	616 surgical ex	316 ex with pathology	300 ex without pathology	cysts, space abscess formation, mandibular fractures		The development of complications is influenced by position of the teeth “position scores”
Zhang [14]	2012	cohort	10–59 n = 1050	Group A: 518 a:17 y Group B: 532 a: 39 y	10–23 n = 518	Group A: 55.5% Group B: 53%	Guangzhou Medical College, China	1050 surgical ex	518 < 23 y	532 > 23 y	orthodontic i.	pathological lesions	Early removal of lower third molar reduce postoperative complications. Group A: 2.48% Group B: 10%
Harradine [7]	1998	RCT	n = 164	14 y		55%		44 surgical ex	44 ex	33 no ex	incisor crowding		No significant difference
Cassetta [13]	2017	cohort	n = 14	Group A: 12.9 ± 0.5 Group B: 12.6 ± 0.5	all	Group A: 50%; Group B: 20%	Sapienza University of Rome, Italy	brass wire ligature with germectomy	brass wire ligature without germectomy		LM2 uprighting		Full eruption LM2 in 5.7 months in both groups; LM3 germ extraction not recommended

n = number, σ = standard deviation; % = percentage, RCT = randomized controlled trial; y = years; a = age; i = indication; LM2 = lower second molar; LM3 = lower third molar.

**Table 2:** Jadad scale for reporting randomized controlled trials.

Jadad Scale for Reporting Randomized Controlled Trials	
Author	Harradine 1998 [7]
1. Is the study described as randomized?	1
2. Is the study described as double blind?	0
3. Is there a description of withdrawals and dropouts?	1
4. The method of randomisation is appropriate?	1
5. The method of blinding is appropriate?	0
6. Total score=	3

1 = Yes; 0 = No.

**Table 3:** Newcastle–Ottawa quality assessment scale for case-control studies.

Newcastle–Ottawa Quality Assessment Scale Case-Control Studies		
	Author	Werkmeister 2005 [8]
**Selection:** (Maximum 4 stars)	1. Is the case definition adequate?	*
	2. Representativeness of the cases	*
	3. Selection of Controls	*
	4. Definition of Controls	*
**Comparability:** (Maximum 2 stars)	5. Comparability of cases and controls on the basis of the design or analysis	*
**Outcome:** (Maximum 3 stars)	6. Ascertainment of exposure	*
	7. Same method of ascertainment for cases and controls	*
	8. Non-Response rate	
	Total score=	7

* The tool is available or described.

**Table 4:** Newcastle–Ottawa quality assessment scale for cohort studies.

Newcastle–Ottawa Quality Assessment Scale Cohort Studies							
Author		Adeyemo 2008 [9]	Chiapasco 1995 [10]	D’Angeli 2021 [11]	Monaco 2016 [12]	Cassetta 2017 [13]	Zhang 2012 [14]
**Selection**: (Maximum 4 stars)	1. Representativeness of the exposed cohort	*	*	*	*	*	*
	2. Selection of the non-exposed cohort	*				*	
	3. Ascertainment of exposure	*	*	*	*	*	*
	4. Demonstration that outcome of interest was not present at start of study	*	*	*			
**Comparability**: (Maximum 2 stars)	5. Comparability of cohorts on the basis of the design or analysis	*			*	*	*
**Outcome:** (Maximum 3 stars)	6. Assessment of outcome **	*	**	**	**	**	*
	7. Was follow-up long enough for outcomes to occur			*	*	*	*
	8. Adequacy of follow up of cohorts			*	*	*	
	Total score =	6	5	7	7	8	5

* The tool is available or described; ** Validated measurement tool.

## 4. Discussion

The aim of this study was to review the literature to assess clinical indications for the germectomy of the third mandibular molar (LM3) in patients at developmental age. This systematic review included eight studies, and based on the results, indications for germectomy were identified, mainly in the field of orthodontics (n = 5) [7,10,11,12,13] and in the field of prophylaxis (to prevent pericoronitis and cyst formation or mandibular angle fracture caused by the LM3) (n = 3) [8,9,14].

### 4.1. Orthodontic Indications

#### 4.1.1. Mandibular Anterior Crowding

In this systematic review, five studies [7,10,11,12,13] have been found to motivate germectomy based on orthodontic indications, such as (a) morphostructural alterations or ectopic impactions, (b) to gain space in the posterior segments of the lower jaw when distalization of first and second molars is necessary, (c) in case of excessive anteroposterior mandibular growth or severe dentoalveolar discrepancy and (d) to prevent relapse after orthodontic therapy, one of which [7] focused on the anterior crowding.

Frontal crowding is a frequently found complaint among orthodontic patients, before and after orthodontic treatment. A study by Robinson et al. in 1859 recognized eruption of a wisdom tooth as the decisive cause of anterior crowding [15]. Furthermore, several studies questioned the cause-and-effect correlation between third molar eruption and anterior crowding [16,17,18].

Scientific literature in this area did not show unambiguous views: Bergstrom and Jensen [19] and Vego [20] were in favor of recognizing the relationship of cause and effect, while Kaplan [21], Ades et al. [22] and Lifschitz and Thilander [23] did not recognize this relationship.

Southard et al. showed that the measures of the contact points after the extraction of wisdom teeth remained unchanged between the two lower dental arches, left and right, with and without extraction, respectively [24]. In addition, Pirttiniemi et al. measured the length of the dental arches on plaster models, immediately and one year after wisdom tooth extraction. His results showed that the extraction of the third molar allowed the distal movement of the second molar, but without affecting the anterior dimension of the arch [25].

This systematic review included the randomized controlled trial by Harradine et al., where anterior crowding was assessed at 66 months after the end of orthodontic therapy in two groups of patients, one of which underwent germectomy and the other did not. The results showed no statistically significant differences between the two groups, with no rationale for a germectomy to prevent anterior segment crowding [7].

Even the most recent studies published after 2010 emphasized the persistence of the dichotomous view. In fact, a study by Esan and Schepartz, published in 2017 [26], which aimed to assess the relationship among LM3 impaction, agenesis and anterior crowding, evaluated mandibles and maxillae of 535 black South African males in the Raymond A. Dart Collection of Human Skeletons, University of the Witwatersrand. The results showed that third molar impaction played a role in anterior crowding as subjects with impaction had more moderate-to-extreme crowding than those with agenesis [26].

By contrary, a study by Cotrin et al. published in 2020 [27], which aimed to evaluate the influence of mandibular third molars on relapse of mandibular anterior crowding in orthodontically treated patients, evaluated the orthodontic records of 108 subjects at three different stages (pre- and post-orthodontic treatment and post retention phase). The authors concluded that the presence or absence of mandibular third molars did not affect the recurrence of anterior mandibular crowding in orthodontically treated patients [27].

Finally, Vasir et al., in a systematic review, showed that the role of the mandibular third molar in late crowding of incisors remains debated. Vasir et al. concluded that the analyzed studies showed a small but statistically significant relationship, and therefore, the clinical findings must be further assessed [28]. In addition, Genest-Beucher et al., in a very recent systematic review aiming to evaluate the impact of the LM3 on mandibular dental anterior crowding, showed that 83% of examined articles did not find any significant relationship between LM3 and mandibular dental anterior crowding [29]. However, the methods and designs of these analyzed studies were questionable.

#### 4.1.2. Lower Second Molar Impaction

One study included in this review correlated LM3 (lower third molar) germectomy and LM2 (lower second molar) impaction [13].

A clinical indication for germectomy is the impaction of LM2 caused by LM3. This indication for germectomy was analyzed, for example, in the study by Vedtofte and Andreasen [30], in which the authors described the incidence of LM2 impaction between 0.6 and 3 every 1000 patients. The authors evaluated the profile radiographs and orthopantomograms from 19 patients with an age range from 8 to 16 years. Although the study group was limited, the results showed that arrested eruption of LM3 occurred more frequently in subjects with a Class II sagittal jaw relationship with a smaller mandibular gonial angle. Interestingly, none of the patients with arrested eruption of LM2 had agenesis of the LM3 [30].

On the contrary, the clinical study by Cassetta et al., which was included in the present qualitative evaluation, aimed to assess the influence of germectomy in a limited group of fourteen subjects with mesially angulated and impacted LM2, who were randomly assigned to a brass wire ligature treatment with or without a germectomy. In both groups, the brass wire technique was effective for LM2 uprighting, while the germectomy group reported a worsening in quality of life after surgery [13].

However, delaying or abstaining from a third molar germectomy, which has already caused angulation and impaction of LM2 requiring orthodontic treatment for uprighting, would always result in the inclusion of the third molar with a potential mesial tilt towards the second molar. In our opinion, the postponement of germectomy may be related to the following clinical problems: 1. the development of caries and periodontal disease affecting LM2; 2. more complex delayed LM3 extraction with possible iatrogenic consequences resulting from root development in the proximity of the mandibular canal.

#### 4.1.3. Molar Distalization

Correction of class II and III malocclusions may require molar distalization that can be achieved through the use of temporary anchorage device (TAD). Molar distalization involves not only proper anchorage, but also the presence of available space distal from the second molar. In this sense, extraction of asymptomatic involved third molars is indicated, even if this movement may be hindered by the displacement of the roots of the second molar within the lingual bone cortex of the mandible [31].

#### 4.1.4. Orthognathic Surgery

The presence of impacted third molars was discussed as a possible cause of an unfavorable postoperative course following mandibular sagittal osteotomy. The indication for early third molar extraction depends on the surgeon’s preferences and orthodontic needs and is scheduled at least 6–9 months prior to orthognathic surgery to allow complete bone maturation at the extraction site [32].

### 4.2. Infectious and/or Cariogenic Causes and Prophylaxis

Three studies included in the present review correlated extraction of LM3 with infectious and cariogenic causes and with prophylaxis [8,9,14].

#### 4.2.1. Infectious and Cariogenic Causes

The study by Adeyemo et al. was conducted in Nigeria between 2001 and 2006, where the authors retrospectively reviewed 1763 patients who had LM3 extraction and the indications for extraction were analyzed [9]. Caries and their complications, recurrent pericoronitis and prophylactic extraction were the major reason for LM3 extraction in 63.2%, 26.3% and 0.6% of the subjects, respectively. It is worth noting that the Nigerian clinical scenario does not reflect the European or American one; moreover, the enrolled subjects were not representative of patients at developmental age uniquely, as the reported age range was 15–92. Young adolescents enrolled in this study were 62 out of 1763 (0.03%) and the vast majority of LM3 extraction was for caries (54.8%) and pericoronitis (45.2%), while orthodontics and prophylactic reasons were not accounted for at all among the youngest group of the study population. The authors concluded that prophylactic extraction is not a common practice in the Nigerian health care system [9].

However, this study cannot reflect the clinical realities of the Western world. Indeed, a descriptive study conducted in Spain on 319 subjects who required LM3 removal showed that prophylaxis was the principal indication of third molar extraction (51.0%), followed by orthodontic reasons [33]. Again, the age of the subjects enrolled in this latter study ranged between 14 and 79 years, highlighting the impossibility to report data on extraction of lower third molars in patients at developmental age, specifically.

#### 4.2.2. Infectious Causes and Prophylaxis

The study included in this review was conducted at the University of Bern over a 5-year period [8]. It analyzed data of 316 patients with a mean age of 33.3 years (range 11–81), who received treatment for deep abscess and cyst formation or mandibular angle fracture in relation to lower LM3 and compared it with a control group of 300 patients with disease-free LM3 removed for prophylactic reasons, in order to assess if these disorders were related to the position of the lower wisdom tooth (angulation, relation to the occlusal plane and to the ascending ramus of the mandible). The conclusions of this study showed that the position of LM3 was a significative risk factor for the development of complications related to unerupted LM3. Moreover, the authors stated that in the cases that present a “high positional risk”, prophylactic removal of LM3 should be justified. Interestingly, this study did not report the enrolled subjects’ characteristics, such as gender and age range, so again, based on the findings of this study, it is impossible to carry out clinical indications for the patients at developmental age [8].

No clinical studies are available on patients at developmental age, and prophylaxis indication to LM3 removal are presented in groups of subjects with very different age range and different-non comparable clinical conditions (LM3 inclusion, LM3 vertical position, LM2 impaction and LM2 and LM3 free and not free from disease). In this scenario, it is not possible to describe evidence-based clinical recommendation. In fact, today, costs of germectomy against non-removal and surgery risks should be evaluated specifically focusing on patients at developmental age. For example, Edwards et al. [34] evaluated the cost, effectiveness and cost effectiveness of removal and retention of asymptomatic, disease free third molars, and they concluded that mandibular third molar retention is less costly to the NHS (National Health Service), more effective for the patient and more cost-effective to both parties than removal. However, should the likelihood of developing pericoronitis, periodontal disease and caries increase substantially then removal becomes the more cost-effective strategy. Interestingly, in the Section 2 of this work, the authors underlined that the decision tree did not include an analysis of differences in surgical morbidity with age or changes in increase of disease with age, because the existing evidence available in relation to these issues is scarce. The authors affirmed that the surgical morbidity probably does not increase with age [34]. The conclusions of Edwards et al. do not agree with the results obtained by D’Angeli et al. [11], who conducted a two-year clinical study after germectomy and showed that postoperative complications after mandibular third molar germectomy during adolescence occur in a significantly reduced percentage of patients, so this oral surgery procedure may become a reliable surgical technique during adolescence [11]. The authors of this review attribute more credibility to D’Angeli’s clinical findings, because he conducted a study focused on germectomy in patients at developmental age. D’Angeli’s findings are in accordance with the study by Chiapasco et al., who showed that the incidence of complications and side effects was 2.6% in the group with an age range from 9–16 years old versus 7.4% in the group older than 24 years old [10].

The management of post-surgical complications after surgical removal of LM3, as periodontal status and increased mobility of LM2, pain, swelling, trismus and temperature has been significantly improved in the last years through the use of novel techniques. Among all the new techniques, it is worth highlighting how kinesiotaping can meet with greater approval, especially in patients of developmental age, as is already known in sports and by the media [35,36,37,38].

The study by Zhang et al. [14] retrospectively evaluated the incidence of complication on a large group of young patients (518), with a mean age of 17 years old, age range 10–23 years, and compared it with a group of 532 adult patients who underwent the same treatment, LM3 extraction. The results of the study showed that the total and nerve complication rate was 2.48% and 0% in the youngest group, compared with 10% and 1.6% in the adult patient’s group, respectively. The authors supported the early removal of LM3, in particularly in the orthodontic patient, but they underlined the importance of a team approach including orthodontist, periodontist, oral surgeon and pediatric dentist for children [14].

Moreover, the growth prevision and the risk of inclusion can be assessed through the evaluation of LM3 position on the orthopanoramic X-ray at the end of the developmental age. Indeed, a recent study by Lauesen et al. confirmed that delayed root development of LM3 at age 15 could be associated with impaction during adulthood [39]. In the context of prophylactic indications to germectomy, LM3 eruption prediction systems are important. These systems can provide information on the possibility of LM3 impaction in adulthood. The analyzed parameters can be skeletal, dental or type of growth [40,41,42,43].

In general, as the patient ages, the complexity of the intervention increases as well, due to greater root development, greater bone density, and thus, a greater risk of iatrogenic damage to the vessels and the nervous system. Pogrel defined the age of 25 as the critical age, beyond which the risk of wisdom tooth extraction increases, also because of the higher risk of infection and periodontal complications distal to the second molar [44].

### 4.3. Limitations

In the analyzed studies, no strict criteria for subject enrollment were present. Age of the enrolled patients ranged from childhood to old age. Moreover, several times, data for the developmental age group were described in the Section 2 of the selected studies, which were then missing in the Section 3, where all the clinical outcomes were described for the whole patient’s group, with data loss regarding the pediatric population.

LM3 extraction is, therefore, described confusingly, as the same assessment criteria cannot be used for adolescent/adult and old subjects, as the entire clinical setting changes, both in terms of tooth development morphology and any complications of surgery.

## 5. Conclusions

Based on the findings of this review, it is not possible to provide evidence-based recommendations for germectomy in Pediatric Dentistry. Therefore, studies focusing on pediatric oral surgery and on germectomy in patients at developmental age, hopefully with a gender-based approach, are awaited in order to present evidence-based criteria for indications for germectomy.

## Data Availability

Not applicable.

## References

[1] Demirjian A., Goldstein H., Tanner J.M. (1973). A new system of dental age assessment. Hum. Biol..

[2] Jung Y.H., Cho B.H. (2014). Radiographic evaluation of third molar development in 6- to 24-year-olds. Imaging Sci. Dent..

[3] Ghaeminia H., Nienhuijs M.E., Toedtling V., Perry J., Tummers M., Hoppenreijs T.J., Van der Sanden W.J., Mettes T.G. (2020). Surgical removal versus retention for the management of asymptomatic disease-free impacted wisdom teeth. Cochrane Database Syst. Rev..

[4] Higgins J., Green S. Cochrane Handbook for Systematic Reviews of Interventions. http://www.cochrane-handbook.org/2011.

[5] Jadad A.R., Moore R.A., Carroll D., Jenkinson C., Reynolds D.J., Gavaghan D.J., McQuay H.J. (1996). Assessing the quality of reports of randomized clinical trials: Is blinding necessary?. Control. Clin. Trials..

[6] Wells G.A., Shea B., O’Connell D., Peterson J., Welch V., Losos M., Tugwell P. (2011). The Newcastle Ottawa Scale (NOS) for Assessing the Quality of Non-Randomised Studies in Meta-Analyses. http://www.ohri.ca/programs/clinical_epidemiology/oxford.asp.

[7] Harradine N.W., Pearson M.H., Toth B. (1998). The effect of extraction of third molars on late lower incisor crowding: A randomized controlled trial. Br. J. Orthod..

[8] Werkmeister R., Fillies T., Joos U., Smolka K. (2005). Relationship between lower wisdom tooth position and cyst development, deep abscess formation and mandibular angle fracture. J. Craniomaxillofac. Surg..

[9] Adeyemo W.L., James O., Ogunlewe M.O., Ladeinde A.L., Taiwo O.A., Olojede A.C. (2008). Indications for extraction of third molars: A review of 1763 cases. Niger. Postgrad. Med. J..

[10] Chiapasco M., Crescentini M., Romanoni G. (1995). Germectomy or delayed removal of mandibular impacted third molars: The relationship between age and incidence of complications. J. Oral. Maxillofac. Surg..

[11] D’Angeli G., Zara F., Vozza I., D’Angeli F.M., Sfasciotti G.L. (2021). The Evaluation of Further Complications after the Extraction of the Third Molar Germ: A Pilot Study in Paediatric Dentistry. Healthcare.

[12] Monaco G., Cecchini S., Gatto M.R., Pelliccioni G.A. (2017). Delayed onset infections after lower third molar germectomy could be related to the space distal to the second molar. Int. J. Oral. Maxillofac. Surg..

[13] Cassetta M., Altieri F. (2017). The influence of mandibular third molar germectomy on the treatment time of impacted mandibular second molars using brass wire: A prospective clinical pilot study. Int. J. Oral. Maxillofac. Surg..

[14] Zhang Q.B., Zhang Z.Q. (2012). Early extraction: A silver bullet to avoid nerve injury in lower third molar removal?. Int. J. Oral. Maxillofac. Surg..

[15] Robinson J. (1859). The causes of irregularities of the teeth. Dent. Rev..

[16] Laskin D.M. (1971). Evaluation of the third molar problem. J. Am. Dent. Assoc..

[17] Lindauer S.J., Laskin D.M., Tüfekçi E., Taylor R.S., Cushing B.J., Best A.M. (2007). Orthodontists’ and surgeons’ opinions on the role of third molars as a cause of dental crowding. Am. J. Orthod. Dentofacial. Orthop..

[18] Tüfekçi E., Svensk D., Kallunki J., Huggare J., Lindauer S.J., Laskin D.M. (2009). Opinions of American and Swedish orthodontists about the role of erupting third molars as a cause of dental crowding. Angle Orthod..

[19] Bergstrom K., Jensen R. (1961). Responsibility of the third molar for secondary crowding. Dent. Abstr..

[20] Vego L. (1962). A longitudinal study of mandibular arch perimeter. Angle Orthod..

[21] Kaplan R.G. (1974). Mandibular third molars and postretention crowding. Am. J. Orthod..

[22] Ades A.G., Joondeph D.R., Little R.M., Chapko M.K. (1990). A long-term study of the relationship of third molars to changes in the mandibular dental arch. Am. J. Orthod. Dentofacial. Orthop..

[23] Lindqvist B., Thilander B. (1982). Extraction of third molars in cases of anticipated crowding in the lower jaw. Am. J. Orthod..

[24] Southard T.E., Southard K.A., Weeda L.W. (1991). Mesial force from unerupted third molars. Am. J. Orthod. Dentofacial. Orthop..

[25] Pirttiniemi P.M., Oikarinen K.S., Raustia A.M. (1994). The effect of removal of all third molars on the dental arches in the third decade of life. CRANIO.

[26] Esan T., Schepartz L.A. (2017). Third molar impaction and agenesis: Influence on anterior crowding. Ann. Hum. Biol..

[27] Cotrin P., Freitas K.M.S., Freitas M.R., Valarelli F.P., Cançado R.H., Janson G. (2020). Evaluation of the influence of mandibular third molars on mandibular anterior crowding relapse. Acta Odontol. Scand..

[28] Vasir N.S., Robinson R.J. (1991). The mandibular third molar and late crowding of the mandibular incisors—A review. Br. J. Orthod..

[29] Genest-Beucher S., Graillon N., Bruneau S., Benzaquen M., Guyot L. (2018). Does mandibular third molar have an impact on dental mandibular anterior crowding? A literature review. J. Stomatol. Oral. Maxillofac. Surg..

[30] Vedtofte H., Andreasen J.O., Kjaer I. (1999). Arrested eruption of the permanent lower second molar. Eur. J. Orthod..

[31] Kim S.J., Choi T.H., Baik H.S., Park Y.C., Lee K.J. (2014). Mandibular posterior anatomic limit for molar distalization. Am. J. Orthod. Dentofacial. Orthop..

[32] Mensink G., Verweij J.P., Frank M.D., Eelco Bergsma J., Richard van Merkesteyn J.P. (2013). Bad split during bilateral sagittal split osteotomy of the mandible with separators: A retrospective study of 427 patients. Br. J. Oral. Maxillofac. Surg..

[33] Fuster Torres M.A., Gargallo Albiol J., Berini Aytés L., Gay Escoda C. (2008). Evaluation of the indication for surgical extraction of third molars according to the oral surgeon and the primary care dentist. Experience in the Master of Oral Surgery and Implantology at Barcelona University Dental School. Med. Oral. Patol. Oral. Cir. Bucal..

[34] Edwards M.J., Brickley M.R., Goodey R.D., Shepherd J.P. (1999). The cost, effectiveness and cost effectiveness of removal and retention of asymptomatic, disease free third molars. Br. Dent. J..

[35] Trybek G., Aniko-Włodarczyk M., Preuss O., Jaroń A. (2021). Assessment of Electrosensitivity of the Pulp of the Mandibular Second Molar after Surgical Removal of an Impacted Mandibular Third Molar. J. Clin. Med..

[36] Trybek G., Rydlińska J., Aniko-Włodarczyk M., Jaroń A. (2021). Effect of Platelet-Rich Fibrin Application on Non-Infectious Complications after Surgical Extraction of Impacted Mandibular Third Molars. Int. J. Environ. Res. Public Health.

[37] Aniko-Włodarczyk M., Jaroń A., Preuss O., Grzywacz A., Trybek G. (2021). Evaluation of the Effect of Surgical Extraction of an Impacted Mandibular Third Molar on the Periodontal Status of the Second Molar-Prospective Study. J. Clin. Med..

[38] Jaroń A., Jedliński M., Grzywacz E., Mazur M., Trybek G. (2020). Kinesiology Taping as an Innovative Measure against Post-Operative Complications after Third Molar Extraction-Systematic Review. J. Clin. Med..

[39] Lauesen S.R., Andreasen J.O., Gerds T.A., Christensen S.S., Borum M., Hillerup S. (2013). Association between third mandibular molar impaction and degree of root development in adolescents. Angle Orthod..

[40] Turley P.K., Joiner M.W., Hellstrom S. (1984). The effect of orthodontic extrusion on traumatically intruded teeth. Am J Orthod..

[41] Turley P.K., Crawford L.B., Carrington K.W. (1987). Traumatically intruded teeth. Angle Orthod..

[42] Ventä I., Murtomaa H., Ylipaavalniemi P. (1997). A device to predict lower third molar eruption. Oral. Surg. Oral. Med. Oral. Pathol. Oral. Radiol. Endodont..

[43] Ventä I. (2012). How often do asymptomatic, disease-free third molars need to be removed?. J. Oral. Maxillofac. Surg..

[44] Pogrel M.A. (2015). Coronectomy: Partial Odontectomy or Intentional Root Retention. Oral. Maxillofac. Surg. Clin. N. Am..

